# Immediate effects of photobiomodulation with low-level laser in women with no laryngeal or voice changes: preliminary results

**DOI:** 10.1590/2317-1782/e20240222en

**Published:** 2025-04-07

**Authors:** Viviane Souza Bicalho Bacelete, Elisa Meiti Ribeiro Lin Plec, Flávio Barbosa Nunes, Andréa Rodrigues Motta, Ana Cristina Côrtes Gama

**Affiliations:** 1 Programa de Pós-graduação em Ciências Fonoaudiológicas, Faculdade de Medicina, Universidade Federal de Minas Gerais – UFMG - Belo Horizonte (MG), Brasil.; 2 Departamento de Oftalmologia e Otorrinolaringologia, Faculdade de Medicina, Universidade Federal de Minas Gerais – UFMG - Belo Horizonte (MG), Brasil.; 3 Departamento de Fonoaudiologia, Faculdade de Medicina, Universidade Federal de Minas Gerais – UFMG - Belo Horizonte (MG), Brasil.

**Keywords:** Larynx, Low-Level Light Therapy, Voice, Rehabilitation, Speech, Language and Hearing Sciences

## Abstract

**Purpose:**

To assess the safety and immediate effect of photobiomodulation of low-level laser in vocally healthy women.

**Methods:**

Experimental research in 36 vocally healthy women aged 18 to 45 years, with skin phototype I to III and body mass index below 25. Participants were randomized to form four groups: Group 1: placebo laser photobiomodulation followed by voiced tongue trill technique (VTTT); Group 2: 3 J infrared laser per point (total 21 J) followed by VTTT; Group: 3: 6 J infrared laser per point (total 42 J) followed by VTTT; and Group 4: 9 J infrared laser per point (total 63 J) followed by VTTT. The following outcomes were assessed: auditory-perceptual evaluation, acoustic analysis (jitter, shimmer, amplitude perturbation quotient [APQ], noise-to-harmonic ratio, period perturbation quotient, cepstral peak prominence, and cepstral peak prominence smoothed), and self-perceived phonatory effort. All participants’ records were taken before and immediately after the experiments.

**Results:**

There was no significant difference in voice quality, acoustic parameters, or self-perceived phonatory discomfort between intervention moments in the placebo, VTTT + 3 J, and VTTT + 6 J groups in the intragroup comparison. G4 (VTTT + 9 J) decreased shimmer and APQ aperiodicity measures (respective p-values: 0.033; 0.044).

**Conclusion:**

Results indicate aperiodicity measures improved with VTTT preceded by 9 J low-level laser application per point, commending this irradiation dosimetry as a possible energy for voice therapy in light-skinned and normal-BMI women. There was no evidence of worsened measures or in-creased discomfort with this resource, indicating it is safe for clinical practice.

## INTRODUCTION

The voice is produced by physiological processes correlated with aerodynamic, mechanical, and acoustic phenomena, resulting from the complex interaction between laryngeal muscles, lower airways, and the vocal tract^(1,2)^. Speech-language-hearing clinical practice widely uses therapeutic approaches to improve muscle performance and treat voice disorders. Hence, research has increasingly addressed the need for specific interventions to elucidate vocal and laryngeal changes in recent years^(3,4)^.

The literature reports a wide range of therapeutic approaches based on exercises, techniques, and programs selected according to therapeutic objectives^(3,4)^ – including voiced trill techniques, among which the voice tongue trill technique (VTTT) stands out with wide clinical applicability^(5)^. To obtain better responses, studies recommend 3-minute exercises in normal-speaking women and 5-minute exercises in cases of dysphonia secondary to vocal nodules^(6,7)^_._

Other approaches – such as photobiomodulation (PBM), a modality that has been advancing in speech-language-hearing therapy – can potentialize therapeutic gain in both habilitation and rehabilitation voice training. PBM is a noninvasive light therapy that uses nonionizing low-level light sources in the visible and infrared spectra – e.g., light-emitting diodes (LEDs), light amplification by stimulated emission of radiation (LASER), and other light sources^(8)^. Once absorbed by chromophores in human cells (particularly, cytochrome c oxidase at the mitochondrial level), the light triggers photophysical and photochemical events on various biological scales^(9)^.

Human skin is a non-homogeneous, highly scattering, and absorbing medium. The physical constituents of the human body, especially fat and melanin concentration, are the mechanisms that influence its optical properties and affect light transmittance through its tissues^(10,11)^.

PBM with low-level laser (LLL) has drawn the attention of health professionals and researchers thanks to its multiple applications and the evidence of positive results in treating various physiopathological conditions with biomodulation. The most cited effects are analgesia^(12)^, inflammatory process modulation^(13)^, healing process acceleration^(14)^, fatigue reduction, and muscle performance improvement^(15)^.

Concerning specifically PBM application in the region of the larynx, studies have investigated the effects of using laser on the vocal folds of humans and animals, finding evidence that suggests modulation of the inflammatory and healing processes in laryngeal tissues^(16,17)^. Only one study assessed the effectiveness of low-level light therapy, irradiating LED to treat vocal fatigue in 16 vocally healthy individuals immediately after a vocal loading task^(18)^. Even though there is a clinical impression of the therapeutic benefits of laser to clinical voice treatment, research is still incipient^(18-22)^, lacking both data on the ideal therapeutic procedure protocol and research demonstrating its effectiveness in dysphonic people.

The literature^(23)^ classifies experimental research on rehabilitation science into five phases. Phase 1 includes single-subject experimental research without the clinical condition to assess the safety and effect of clinical intervention doses and studies are important to foment further studies with higher levels of evidence. Hence, this study was based on the following research question: “What is the effect and safety of different infrared LLL doses (3, 6, and 9 Joules) combined with VTTT on the quality and self-perception of voice in vocally healthy women?”.

This research is justified as it presents scientific knowledge on the safety and immediate effects of different LLL doses, providing the basis for further research on the effectiveness of this therapeutic approach in voice rehabilitation, designed to have higher levels of evidence, such as randomized clinical trials.

## METHODS

This randomized experimental intrasubject comparison study (Phase 1)^(23)^ was approved by the Ethics Committee of the Federal University of Minas Gerais (UFMG) (4.704.038).

The research sample comprised 36 women aged 18 to 45 years with neutral voice quality, without vocal/laryngeal complaints or symptoms, with skin phototypes I to III according to the Fitzpatrick scale^(24)^, and body mass index (BMI) below 25. All participants performed the VTTT^(25)^.

The study was carried out at the Observatory of Speech-Language-Hearing Functional Health of the UFMG Medical School and the Otorhinolaryngological Service at the UFMG Clinics Hospital. The volunteers were informed of the research objectives and procedures, read the informed consent form, had their questions answered, and signed the form. They were recruited as personally invited university students and employees and announced the research on social media, characterizing it as a convenience sample.

Participants who had a self-perceived positive voice quality (good or very good voice) and did not have voice symptoms (fatigue and/or discomfort) were invited to undergo speech-language-hearing and otorhinolaryngological assessment for sample selection. The speech-language-hearing assessment was made by one of the researchers with more than 10 years of experience in auditory-perceptual evaluation. It included the analysis of the grade of hoarseness (G) on a 4-point scale (neutral, mild, moderate, and intense), with tasks of habitual sustained vowel /a/ and linked speech (days of the week). The otorhinolaryngological assessment was conducted by a single otorhinolaryngologist, with flexible fiberoptic nasolaryngoscopy. Laryngeal examination results were considered normal when they revealed full glottal closure and no vocal fold lesions. The presence of posterior glottic chink was considered physiological^(26)^.

The study included participants with a neutral voice quality (G0) and no laryngeal lesions. The exclusion criteria were pregnant women; women with suspected pregnancy; in the premenstrual period, with an allergic reaction, and/or respiratory condition on the day of the assessment; with a systemic, neurological, and/or neoplastic disease; smokers; women who had been previously submitted to speech-language-hearing and/or surgical treatment due to voice changes; who were photosensitive or had a skin disease/lesion; who had a tattoo in the region where light would be applied; and those who reported taking skin treatment medications.

The participants’ skin phototypes were defined based on their self-assessed sensitivity to the sun, considering six skin color phototypes ranging from fair (type I skin) to darkest brown (type VI skin) on the Fitzpatrick scale^(24)^.

BMI was calculated by dividing the body mass by the square of the body height. The participants were weighed and measured by the main researcher. Hence, the women’s nutritional statuses were classified based on the cutoff scores established in the literature (BMI ˂ 25: normal weight; BMI ≥ 25: overweight)^(25)^.

The groups were matched for age (p = 0.095), BMI (p = 0.103), and skin phototype (p > 0.05). The participants’ mean age was 28.5 years (minimum = 18; maximum = 45; SD: 6.9) and their mean BMI was 21.3 (minimum: 17; maximum: 24.5; SD: 2.7). On the Fitzpatrick scale, 52.3% of the participants had a white skin (types I and II), and 47.3% had a golden honey skin (type III).

Participants were randomly allocated in blocks to form even groups with a fixed number of individuals, according to their treatments. The researchers organized envelopes with papers numbered 1 to 4; each envelope represented a randomization block, and each block was randomly defined in a draw, allocating them to the experimental and placebo groups. The participants were blinded to this procedure.

The randomization and allocation process distributed all participants blindly into four equal groups, with nine of them in each one:

**Group 1 (G1)** – Application of placebo infrared laser (the equipment emitted light but not energy) for 60 seconds, immediately followed by VTTT for 3 minutes^(6)^. The anatomical points were outlined for PBM application as in experimental group procedures, but the equipment was manipulated to sound a beep without emitting therapeutic light;
**Group 2 (G2)** – Application of infrared laser for 30 seconds in 3 J doses per point^(19)^, totaling a 21 J dose in the larynx. Immediately after applying the laser, participants performed VTTT for 3 minutes^(6)^;
**Group 3 (G3)** – Application of infrared laser for 60 seconds in 6 J doses per point^(19)^, totaling a 42 J dose in the larynx. Immediately after applying the laser, participants performed VTTT for 3 minutes^(6)^;
**Group 4 (G4)** – Application of infrared laser for 90 seconds in 9 J doses per point^(19)^, totaling a 63 J dose in the larynx. Immediately after applying the laser, participants performed VTTT for 3 minutes^(6)^.

### Photobiomodulation

The laser was applied with equipment manufactured by DMC, model Therapy EC, with 100 mW power, spot measuring 0.0984 cm^2^, and infrared wavelength (808 ± 10 nanometers), either placebo or with 3 J, 6 J, or 9 J doses, always before VTTT.

The dosimetry parameters are described in detail in Table 1.

**Table 1:** Photobiomodulation parameters with low-level laser

Dosimetric parameters	Placebo	G2	G3	G4
		(3 J+VTTT)	(6 J+VTTT)	(9 J+VTTT)
Wavelength (nm)	NA	808 ± 10	808 ± 10	808 ± 10
Equipment power (mW)	NA	100	100	100
Output spot (cm^2^)	NA	0.0984	0.0984	0.0984
Irradiance or power density (W/cm^2^)	NA	1.01	1.01	1.01
Flow or energy density (J/cm^2^)	NA	30.5	61	91.5
Emission mode	NA	continuous	continuous	continuous
Irradiation application mode	NA	point	point	point
Energy per point (J)	NA	3	6	9
Total irradiated points	NA	7	7	7
Irradiation time per point (s)	NA	30	60	90
Total energy (J)	NA	21	42	63

Caption: cm^2^ = square centimeter; J = joule; J/cm^2^ = joule per square centimeter; G2 = group 2; G3 = group 3; G4 = group 4; VTTT = voiced tongue trill technique; mW = milliwatts; NA = not applicable; nm = nanometers; W/cm^2^ = power per square centimeter

**Figure 1:**
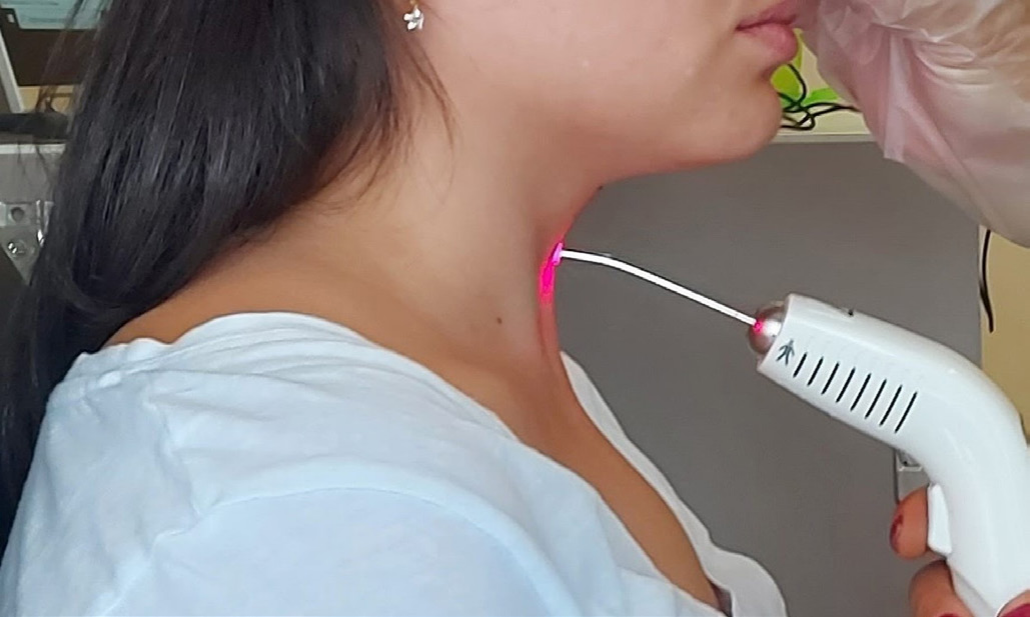
Low-level laser application on the larynx

Participants were sitting on a chair during the application, with their heads in a neutral position. Their skin was cleaned before beginning therapeutic light irradiation, rubbing the neck with 70% alcohol. Then, the tip of the equipment was wrapped with PVC film. The laryngeal region received continuous point application – i.e., the tip of the equipment was in direct perpendicular contact with the skin (Figure 1).

The same researcher applied the LASER to all groups, having been trained to locate the anatomical points and use the therapeutic resource.

The anatomical limits were established for irradiation, defining the approximate location of the glottic level and the main intrinsic laryngeal muscles, based on previous research, in which the speech-language-hearing therapist palpated the participants’ neck structures^(19)^. Seven points (one central and three in each hemilarynx) were identified to apply therapeutic light to the neck region, according to the anatomical references established in the previous study^(19)^. **Point 1** was defined as the midpoint between the laryngeal incisure and the lower border of the thyroid cartilage, in the topography of the anterior commissure of the larynx; **point 2** was located 1 cm away from the midpoint, in the region of the TA muscle; **point 3** was located 1 cm away from point 2, aiming at the LCA muscle; lastly, **point 4** was defined as the point in the cricothyroid space 1.5 cm away from the midpoint, in the topography of the CT muscle^(19)^.

**Figure 2:**
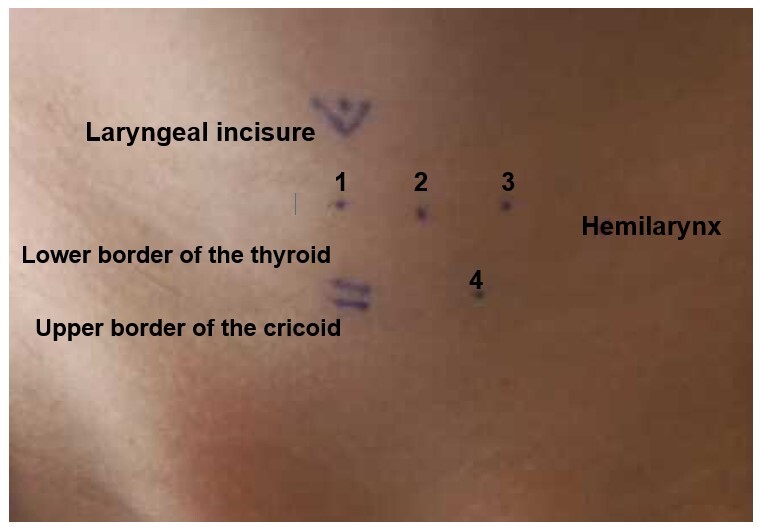
Schematic representation of the laser application points in the larynx

Figure 2, below, shows the anatomical points delimited for LLL application in the larynx.

Caption: (1) Point 1 (anterior commissure of the larynx); (2) Point 2 (membranous portion of the vocal fold – TA); (3) Point 3 (cartilaginous portion of the vocal folds – LCA); (4) Point 4 (CT muscle)

### Voiced tongue trill technique

The participants stood upright to perform the VTTT for 3 minutes at their usual, comfortable, average pitch, without anterior head or jaw movement. The researcher monitored their rhythm and breathing^(6)^, timed the exercises with a digital stopwatch, and counted the number of exercise repetitions per period.

### Assessment of the outcome variables

**Figure 3:**
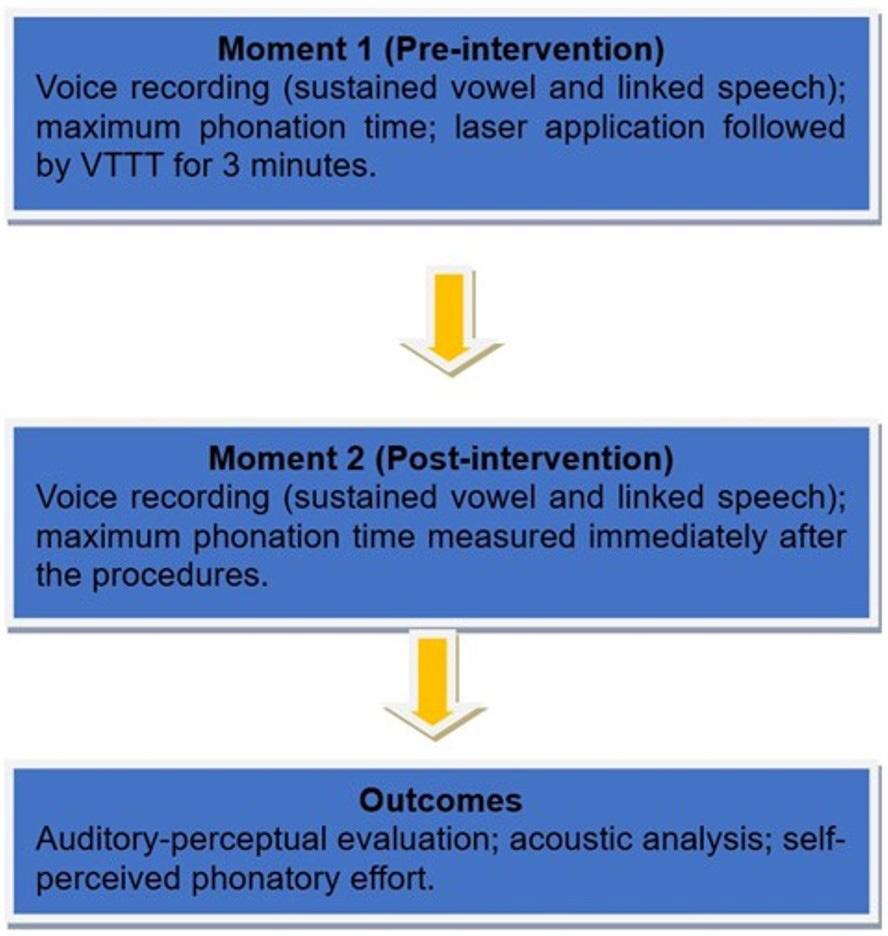
Flowchart of the study stages

The following dependent variables were considered to analyze the immediate LLL effect on normal-speaking women: 1) auditory-perceptual evaluation of voice quality; 2) acoustic analysis of voice; and 3) self-perceived phonatory effort. The outcome variables were assessed both before (moment 1) and immediately after the intervention (moment 2) in all groups. The procedures were carried out in a single session (Figure 3).

Caption: VTTT: voiced tongue trill technique

All collection procedures of the dependent variables are detailed below.

#### Voice recording and auditory-perceptual evaluation

Voice samples were obtained with a unidirectional condenser microphone manufactured by Shure^®^, positioned at 45º in front of the mouth, 4 cm away from the corner of the mouth, and recorded directly in a computer system (Dell computer, model Optiplex GX260, with a professional sound card manufactured by DirectSound^®^). They were recorded in an acoustically treated room, and the voices were edited in Audacity program, 2.0.6. Volunteers stood throughout the recording and emitted a sustained vowel /a/ in habitual pitch and loudness.

The auditory-perceptual evaluation of voice quality was performed by five speech-language-hearing therapists with more than 10 years of experience in such evaluations, blinded to the intervention and assessment moment. The pairs of voices named A or B were randomized, not revealing the moment of the intervention. Evaluators were instructed to rest their ears for 15 minutes every 22 voices to minimize the risk of response errors due to fatigue^(6)^.

Evaluators were instructed to consider whether one of the voices in each pair was better than the other (A or B) or whether their overall voice quality was the same. Responses were categorized and tabulated as follows:

If the voice after the experiment was considered better = improved;
If the voice before the experiment was considered better = worsened;
If the voices were considered equal = unchanged.

The intrarater agreement in the auditory-perceptual evaluation was assessed with the Gwet AC1 statistics in R software, version 3.3.1. The degree of agreement was analyzed as follows: values below zero – no agreement; from 0 to 0.20 – small agreement; from 0.21 to 0.40 – weak agreement; from 0.41 to 0.60 – moderate agreement; from 0.61 to 0.80 – good agreement; and from 0.81 to 1.00 – almost perfect agreement^(27)^. The evaluators’ degrees of agreement were respectively 47%, 50%, 68%, 73%, and 82%. Thus, the responses of the three evaluators who had a good or almost perfect agreement were considered for the auditory-perceptual evaluation of voice, using the mode of the three judges’ responses.

#### Analysis of the acoustic measures

The voices were recorded using the Computerized Speech Lab (CSL) program by Kay Pentax^®^, model 6103, Multi-Dimensional Voice Program (MDVP) module^28^, installed in a Dell^®^ computer, model Optiplex GX260, with a professional soundcard manufactured by DirectSound^®^, and a unidirectional condenser microphone manufactured by Shure^®^.Participants stood in front of the microphone, which was placed on a pedestal at mouth height, 4 cm away from the mouth. They were instructed to prolong the vowel /a/ emission habitually and fully and count from 1 to 20. All recordings were made in an acoustically treated room.

The following parameters were used for acoustic analysis:

1. Fundamental frequency (f_0_): mean of all extracted frequency periods. The program’s handbook indicates 243.97 Hertz (Hz) as the normal value for women^(28)^;
2. Jitter and pitch perturbation quotient (PPQ): parameters that measure short-term pitch perturbation, presented in percentages – the normal value for women is 0.36%^(28)^;
3. Shimmer and amplitude perturbation quotient (APQ): parameters that measure short-term amplitude perturbation, presented in percentages – the normal value for women is 1.39%^(28)^;
4. Noise-to-harmonic ratio (NHR): a measure that relates harmonic to noise in the acoustic wave. The normal value for women is 0.11 dB^(28)^;
5. Maximum phonation time: participants were instructed to emit a sustained vowel (/a/) while sitting comfortably on a chair in an acoustically treated room. The normal time for women is 15 to 25 seconds^(29)^;
6. Cepstral measures: these were taken with the Praat program, version 6.0.52, selecting the best, middle 3-second excerpt of the sustained vowel /a/ emission, dismissing its beginning and end, as well as the full linked speech emission. The cepstral peak prominence (CPP) and cepstral peak prominence smoothed (CPPS) were extracted from the vowel /a/ and linked speech, selecting the parameters proposed in the literature^(30)^.

#### Analysis of self-perceived phonatory effort

The self-perceived phonatory effort was analyzed with the Borg Scale CR10-BR, in which 0 indicates no vocal effort and 10 indicates maximum effort. After the procedure, subjects respond to it on a Likert-type scale, as follows: 0 “No phonatory effort”; 0.5 “Very slight, barely perceivable vocal effort”; 1 “Very mild vocal effort”; 2 “mild vocal effort”; 3 “Moderate vocal effort”; 4 “Somewhat severe vocal effort”; 5 “Severe vocal effort”; 6, 7, and 8 “Very severe vocal effort”; 9 “Extremely severe, almost maximum vocal effort”, and 10 “maximum vocal effort”^(31)^.

The sustained vowel emission task was used as a reference for analyzing self-perceived phonatory effort before and after the procedures.

### Statistical analysis

Data were statistically analyzed with the MINITAB statistical program, version 17. The distribution of quantitative variables analyzed with the Anderson-Darling test defined the statistical paired t-test for intragroup parametric analysis before and after the experiment (Placebo group: jitter, shimmer, APQ, PPQ, CPP, and CPPS vowel and speech; Group 3 J: F0, jitter, MPT, shimmer, PPQ, APQ, NHR, CPP, and CPPs vowel and speech; Group 6J: MPT, F0, shimmer, APQ, CPP, and CCPs vowel and speech; Group 9 J: MPT, jitter, APQ, PPQ, CPP, and CCPs vowel and speech). The Wilcoxon test was used for the intragroup analysis of data without a normal distribution (Placebo group: F0 and NHR; Group 3 J: jitter and PPQ; Group 9 J: F0, shimmer, and NHR). Categorical variables were statistically analyzed with the chi-square test. In all analyses, the level of significance was set at 5%.

## RESULTS

The mean number of tongue trills was 23 in G1 (placebo), 29 in G2 (VTTT + 3 J), 26 in G3 (VTTT + 6 J), and 23 in G4 (VTTT + 9 J) with no difference between the groups (p = 0.504).

Table 2 presents the auditory-perceptual evaluation results comparing the groups before and after the procedures. There was a greater occurrence of unchanged voice quality after the experiments, with no statistical significance between the groups.

**Table 2:** Comparison of auditory-perceptual evaluation results between groups

Voice quality	G1 (placebo)		G2 (3 J+VTTT)		G3 (6 J+VTTT)		G4 (9 J+VTTT)		Total		*p-value
	n	%	n	%	n	%	n	%	n	%	
Improved	1	11	1	11	2	22.3	1	11	5	13.9	0.985
Worsened	2	22.3	2	22.3	1	11	2	22.3	7	19.4	
Unchanged	6	66.7	6	66.7	6	66.7	6	66.7	24	66.7	

* Chi-square test

Caption: G = group; J = joule; n = number; VTTT = voiced tongue trill technique

Table 3 shows the results of the intragroup comparison before and after the intervention. They reveal no acoustic changes in the groups after VTTT alone or in combination with 3 and 6 J. However, the group that used VTTT in combination with 9 J had a significant decrease in amplitude aperiodicity measures (shimmer and APQ).

**Table 3:** Acoustic measures before and after VTTT alone (placebo laser) and VTTT in combination with 3 joules, 6 joules, and 9 joules of energy

Parameters			VTTT alone (placebo laser)			VTTT combined with 3-J energy			VTTT combined with 6-J energy			VTTT combined with 9-J energy		
			Before	After	Pre/post p-value	Before	After	Pre/post p-value	Before	After	Pre/post p-value	Before	After	Pre/post p-value
**MPT (s) /a/**		mean	11	10.7	0.590	10.55	11.77	0.193	12	13.8	0.332	10.8	10.7	0.942
		median	10	10		10	11		10	13		10	9	
		minimum	8	6		9	9		9	9		8	6	
		maximum	18	18		14	15		18	22		14	18	
		SD	3.27	3.095		1.74	2.048		3.162	4.676		2.261	3.898	
**F0 (Hz)**		mean	214.1	210.74	0.772	196.87	198.87	0.830	197.49	199.82	0.693	201.88	210.22	0.058
		median	223.22	216.57		199.82	205.75		201.25	195.39		193.77	208.95	
		minimum	175.15	164.32		174.7	156.64		180	186.02		183.5	184.99	
		maximum	234.85	248.84		214.95	225.94		215.03	218.3		259.48	266.46	
		SD	20.79	27.06		14.67	23.16		12.58	11.96		23.2	23.43	
**Jitter (%)**		mean	1.135	1.072	0.810	1.126	1.155	0.920	1.1337	1.4028	0.076	1.2113	1.097	0.695
		median	1.104	1.055		1.1	1.114		0.906	1.219		1.113	1.183	
		minimum	0.387	0.640		0.273	0.56		0.541	0.706		0.414	0.518	
		maximum	2.542	1.669		2.603	2		2.197	2.477		2.476	1.727	
		SD	0.680	0.338		0.67	0.5		0.63	0.673		0.734	0.435	
**Shimmer (dB)**		Mean	3.459	3.424	0.926	3.886	3.581	0.588	3.9	3.937	0.936	4.49	3.5	**0.033** *
		median	3.443	3.303		3.641	3.378		4.238	3.881		4.17	3.43	
		minimum	2.2	2.916		1.748	2.26		2.222	2.786		2.889	0.336	
		maximum	4.907	4.064		5.32	5.97		5.21	5.37		7.95	5.716	
		SD	1.007	0.399		1.2	1.12		1.019	0.888		1.398	1.447	
**PPQ (%)**		mean	0.658	0.625	0.828	0.657	0.654	0.984	0.656	0.818	0.058	0.694	0.617	0.629
		median	0.643	0.615		0.67	0.65		0.529	0.724		0.644	0.688	
		minimum	0.229	0.362		0.158	0.324		0.329	0.424		0.263	0.326	
		maximum	1.495	0.981		1.53	1.15		1.25	1.46		1.443	0.952	
		SD	0.394	0.206		0.394	0.287		0.354	0.395		0.417	0.216	
**APQ (%)**		mean	2.364	2.347	0.942	2.67	2.42	0.520	2.818	2.7169	0.775	3.036	2.677	0.044*
		median	2.401	2.277		2.49	2.26		2.844	2.62		2.916	2.646	
		minimum	1.53	1.971		1.26	1.56		1.57	1.939		2.012	1.999	
		maximum	3.23	2.804		3.69	3.965		4.42	3.625		5.126	3.98	
		SD	0.639	0.281		0.838	0.761		0.856	0.611		0.867	0.592	
**NHR (dB)**		mean	0.118	0.128	0.244	0.136	0.118	0.088	0.132	0.125	0.445	0.1211	0.125	1
		median	0.124	0.158		0.136	0.126		0.134	0.129		0.126	0.112	
		minimum	0.08	0.110		0.109	0.072		0.111	0.096		0.09	0.104	
		maximum	0.143	0.154		0.16	0.146		0.153	0.15		0.149	0.197	
		SD	0.021	0.013		0.014	0.024		0.014	0.018		0.02	0.029	
**CPP (dB)**		Mean	21.6	21.15	0.769	23.16	22.48	0.701	23.6	23.5	0.960	22.97	22.92	0.979
		median	20.65	20.55		21.7	23		23.6	24		23.5	23.75	
	vowel	minimum	16.29	14.9		18.7	18		18.9	18.5		16.4	16.3	
		maximum	26.15	28.5		29.5	29.15		28.6	28.8		29.35	29	
		SD	3.129	4.059		3.835	3.5		3.312	3.19		3.866	3.75	
		mean	14.84	14.75	0.924	14.41	14.09	0.612	15.5	15.6	0.919	15.1	15.73	0.617
		median	14.55	13.75		14.5	14.15		15.15	15.35		15	15.8	
	speech	minimum	12.3	12.3		12.25	12.7		13.7	13.8		12.5	12.7	
		maximum	17.2	17.6		16.9	15.3		18.9	17.8		20.4	20	
		SD	2.02	2.07		1.521	1.073		1.56	1.244		2.524	2.712	
**CPPS (dB)**		mean	13.13	12.83	0.767	14.12	12.6	0.357	14.252	14	0.910	12.95	13.88	0.545
		median	13.1	12.35		13.35	13.4		15.2	15.6		13.7	14.4	
	vowel	minimum	10.25	9.85		11.25	4.25		9.15	9.3		7.6	8.7	
		maximum	16	17.5		19.3	18.2		18.45	18		18.3	18.4	
		SD	1.87	2.418		2.97	3.74		3.181	3.056		3.39	2.98	
		mean	6.33	6.35	0.982	5.79	5.39	0.531	6.5	6.73	0.782	6.78	7.27	0.678
		median	6.35	5.5		5.25	5.5		5.9	6.6		6.9	6.9	
	speech	minimum	4.4	4.5		4	4.25		4.63	4.3		4.4	4.7	
		maximum	8.2	8.85		8.3	6.8		8.7	9.2		11.7	11.45	
		SD	1.45	1.581		1.53	1.06		1.59	1.82		2.35	2.53	

* p<0.05

**Caption:** APQ = amplitude perturbation quotient; CCP = cepstral peak prominence; CPPS = cepstral peak prominence-smoothed: dB = decibel; F0 = fundamental frequency; SD = standard deviation; Hz = hertz; NHR = noise-to-harmonic ratio; PPQ = pitch perturbation quotient; s = seconds; MPT = maximum phonation time; VTTT = voiced tongue trill technique

The self-perceived effort was reported after the experiment in the placebo group (G1) (n = 2) and experimental groups G2 (n = 1) and G4 (n = 4), though with no statistical difference between the groups, according to the results shown in Table 4. As for the level of phonatory effort verified with the Borg Scale CR10-BR^(31)^, most participants (80.5%) did not feel it worsened during voice production after the procedures. Concerning the participants who perceived some effort (19.5%), most of them (85.7%) reported it at level 1 (very mild vocal effort) while only one subject (14.3%), who was from the placebo group, reported it at level 2 (mild vocal effort).

**Table 4:** Vocal effort evaluation result in comparison between groups

Vocal effort	G1 (placebo)		G2 (3J+VTTT)		G3 (6J+VTTT)		G4 (9J+VTTT)		Total		*p-value
	n	%	n	%	n	%	n	%	n	%	
present	2	22.3	1	11.2	0	0	4	45.5	7	19.5	0.063
absent	7	77.7	8	88.8	9	100	5	55.5	29	80.5

* Chi-square test

Caption: G = group; J = joule; n = number; VTTT = voiced tongue trill technique

## DISCUSSION

Voice therapy consists of specific exercises to control and coordinate different aspects of voice production^(1,2)^. This study chose VTTT as an experimental treatment after LLL irradiation due to its wide application in voice clinical practice and its benefits in attenuating the contact between vocal folds and changing vibration patterns^(6,7)^. The performance lasted 3 minutes, as recommended for normal-speaking women^(6)^.

Activating oral, laryngeal, and thoracic muscles with VTTT consumes much energy, furnished by cell metabolism^(6)^. According to the principle of applying light to increase energetic metabolism, PBM in the intrinsic laryngeal musculature before exercise may potentialize therapeutic gains because bioenergetic pathways in the muscles provide high mitochondrial content with specialized functional demands of the larynx^(9)^.

The lack of specification details regarding laser irradiation parameters and the wide range of experimental methods limit the comparison of results and the replication of benefits in new experimental studies, also making the replication of protocols unfeasible in professional clinical practice^(32)^.

The results of the auditory-perceptual evaluation revealed that most participants’ voices remained unchanged after VTTT alone or in combination with different laser doses, with no statistical differences between the groups. It is possible that the findings did not demonstrate an improvement in voice quality because study participants had neutral voice quality and the auditory-perceptual evaluation was not sensitive to perceive subtle changes in voice production^(33)^. Another study likewise did not find voice improvement after 3 minutes of VTTT^(34)^.

The acoustic analysis results did not find significant differences in any of its analyzed parameters before and after VTTT in the placebo group – i.e., alone. Similar results were found in studies that demonstrated that f_0_, jitter, shimmer, NHR, and CPPS were not sensitive to assess immediate VTTT effects in normal-speaking subjects^(33,34)^. The combination of VTTT with 3 J and 6 J laser revealed no significant effect immediately after the treatment on the auditory-perceptual or acoustic measures. Similarly, a previous study did not find effects on acoustic, auditory-perceptual, and self-perceived voice production parameters after four sessions of 6 J laser over 1 month^(20)^. However, it applied laser alone, which may be why no changes were demonstrated in muscle adjustments after repeatedly applying this therapeutic resource^(20)^. Another study also did not demonstrate auditory-perceptual or acoustic changes after immediately applying LED, although it found positive results 1 hour after intervention^(18)^. Other authors likewise did not find an immediate effect of 6 J PBM in combination with VTTT in amateur singers^(22)^.On the other hand, VTTT in combination with 9 J energy per point had a trend (without statistical significance) toward increasing f_0_, decreasing frequency aperiodicity measures (jitter and PPQ), and increasing CPPS measures, besides a statistically significant difference in amplitude aperiodicity measures (shimmer and APQ).

Among the assessed acoustic parameters, increased f_0_ may be associated with repetitive TA contraction during the exercise. The literature demonstrates that semi-occluded vocal tract exercises result in muscle and functional laryngeal adjustments, replacing LCA activity with greater TA activity^(6,35)^. Decreased PPQ measures may suggest greater vibration regularity in the vocal folds^(29)^. Increased CPPS indicates an increased harmonical structure of the voice signal because the cepstral peak is the acoustic energy that overshadows the background noise and may be related to improvements in the mucosal wave^(36)^. The results in this research indicate that 9 J LLL application in combination with VTTT decreases amplitude aperiodicity measures, improving vocal fold vibration periodicity – which suggests that this is the ideal application dose.

The self-assessment revealed no difference in the perception of greater phonatory effort after laser application in the study groups. The absence of significantly worse effort sensation in voice production after applying irradiation suggests that this resource can be safely used in clinical practice^(20)^. However, it is not possible to say that repeated applications of PBM in the larynx are free of cell damage in the medium and long term^(37)^. Vocal self-assessment has been highly valued and is useful for evaluating the impact of the deviation on the patient's life, monitoring progress, assessing the effectiveness of the treatment, and playing an important role in therapeutic decisions. The Borg CR10-BR Scale adapted for vocal effort is a specific self-assessment instrument for vocal effort after specific tasks^(31)^.

Applying a 9 J infrared laser in seven laryngeal points may be promising dosimetry to potentialize the effects of vocal exercises. However, the sample size and laser effect assessment time immediately after exercises are limitations of this research. It is necessary to know light delivery parameters to the target tissues to optimize treatment – which poses the challenge of quantifying doses based on factors that interfere with the biophysical capacity of light penetrating the skin and reaching structures in a certain depth^(10,11)^. Thus, this research controlled variables that can interfere with tissue penetration (such as sex, age, physiological status of the tissue, and skin pigmentation and thickness), and the homogeneous groups regarding these characteristics were randomly distributed to diminish the risk of selection bias. Although the selection of a sample with rather specific characteristics was a limiting factor of this study regarding sample size, we believe that the correct prescription of irradiation parameters must be individualized and adjusted to personal characteristics.

The translational research movement aims to bring scientific knowledge closer to the clinical routine. Hence, the analysis of the immediate effect of PBM on the voice helps develop clinical protocols, identify best practices, and ground future research with higher levels of evidence. It is fair to assume that investigating therapeutic light application with immediate assessment is not enough to verify cell changes capable of changing muscle patterns and immediate objective responses. Muscle adjustments and voice changes may take longer to occur. We emphasize that this study presents preliminary results regarding the immediate effects of LLL in women, but we still don't know the short and medium-term effects in different conditions in the voice clinic. Elucidating the time needed to increase the biochemical and biomodulatory effects of cellular functions with potentiation of therapeutic gains requires specific training programs that also consider exercise physiology, as well as repeated applications of therapeutic light with longitudinal monitoring of vocal responses at different times after irradiation. Hence, programs with repeated applications and longitudinal follow-up to assess the cumulative PBM effect or analyze vocal responses hours after laser application may elucidate the time necessary for the biochemical increase and biomodulator effects on cell functions, potentializing therapeutic gains.

Finally, as this study had a limited sample size and an immediate effect analysis, future research should encompass larger samples, assessing vocal responses hours and days after irradiation, whose samples have different ages, sexes, and skin pigmentation and thickness characteristics, and with other designs (such as randomized clinical trials) to add further knowledge on LLL application in voice clinical practice.

The clinical reasoning behind PBM recommendation in the area of voice is based on knowledge obtained from related areas and the experience of professionals who witness potentialized therapeutic gains^(12-14)^. However, recommendation criteria to apply LLL must be grounded on evidence-based practice guidelines, which include not only experts’ opinions but also evidence from scientific research and the patient’s values and preferences. There are still gaps regarding the irradiated area (density of applied energy), time-response (how long before the exercise protocol the light must be applied, and how long after it the responses occur), and the irradiated energy (energy dose per application point). Hence, professional practice must be given support with guidance in various topics to better define voice clinical procedures.

## CONCLUSION

PBM therapy with an infrared laser before exercise improved shimmer and APQ after applying 9 J per point in seven laryngeal points in the experimental group. This may be an ideal irradiation dosimetry, within a therapeutic window recommendable for clinical vocal practice in light-skinned women with normal BMI and no voice or laryngeal changes. In addition, the comparison of results obtained with VTTT alone or associated with LLL did not reveal any significant worsening in overall voice quality or any difference between the groups in the perception of phonatory effort during sustained vowel emission, suggesting that this therapeutic resource does not cause any vocal damage in the immediate effect analysis.

However, the results should be carefully interpreted due to the sample size and immediate effect analysis. Future studies with larger samples and analysis of different time windows after applying therapeutic light are needed to analyze the short-, medium-, and long-term effects on both vocal quality and self-perception of phonatory effort to affirm the safety of applying this resource in the vocal clinic.
